# Dietary protein intake and asthma in US children and adolescents: a cross-sectional analysis of NHANES data

**DOI:** 10.1016/j.jped.2025.101495

**Published:** 2026-01-06

**Authors:** Lingyu Li, Peipei Tian, Yanmei Teng, Rui Wang, Mingxiao Zhang, Qiuyu Ma

**Affiliations:** People's Hospital of Cangzhou, Cangzhou, China

**Keywords:** Asthma, Protein, Child, Adolescent, Cross-sectional study

## Abstract

**Objective:**

Evidence regarding total dietary protein intake remains inconsistent and limited for the US pediatric population. This study utilizes nationally representative data to examine the association between total dietary protein intake and asthma prevalence, thereby addressing this research gap.

**Method:**

A cross-sectional study that involved 4825 people used data from the National Health and Nutrition Examination Survey (NHANES) from 2011 to 2020. The relationship between total protein intake and asthma was investigated using multiple linear regression models. The linear relationship between the two was tested using a restricted cubic spline. Stratified analysis further confirms the stability of the findings.

**Results:**

All eligible participants (N = 4,825; mean age 10.6 ± 2.9 years; 49.7% male) were analyzed. The fully-adjusted model revealed a positive association between total protein consumption and asthma after controlling for confounders. Compared to the lowest intake quartile Q1 (≤ 3.966 mg/day), adjusted odds ratios for asthma in Q2 (3.967-6.364 mg/day) and Q3 (≥ 6.365 mg/day) were 0.097 (95%CI:0.97-1.44, p = 0.097) and 0.036 (95%CI:1.01-1.55, p = 0.036), respectively. A dose-response relationship (p = 0.037) emerged between total protein consumption and asthma risk.

**Conclusion:**

Total dietary protein intake demonstrated a positive association with asthma among US children and adolescents. These findings are statistically significant.

## Introduction

Asthma remains the most prevalent chronic disease among children and adolescents in the United States, imposing a substantial public health burden. Current estimates indicate that approximately 5.5 million children under 18 years (7.5 %) are affected, with annual costs exceeding $80 billion, driven by direct medical expenditures and indirect costs related to lost productivity^1^, characterized by recurrent episodes of wheezing, breathlessness, chest tightness, and coughing, significantly impacting quality of life, school attendance, and healthcare utilization.^2^,^3^ While asthma incidence and mortality rates have plateaued in recent years, profound disparities persist, disproportionately affecting marginalized populations, including racial/ethnic minorities and children from low socioeconomic backgrounds.^4^ The complex etiology involves intricate interactions between genetic predisposition and diverse environmental exposures. Among modifiable environmental factors, dietary patterns have emerged as a significant area of investigation, potentially influencing immune development, airway inflammation, and lung function trajectories.^5^

However, Airway remodeling closely tracks disease progression, culminating in progressive lung function decline. Despite advances in pharmacotherapy, current interventions fail to modify the natural history of asthma. Past research has predominantly examined the relationship between nutritional factors and airway inflammatory processes. The potential role of diet in asthma pathogenesis has garnered considerable scientific interest.^6^,^7^ Extensive research has explored associations between asthma outcomes and various dietary components, including antioxidants (vitamins C, E), minerals (magnesium, selenium), fatty acids (omega-3/omega-6 ratio), fruit/vegetable intake, and processed foods.^8^,^9^ While findings are sometimes inconsistent, systematic reviews suggest protective effects for Mediterranean-style diets rich in fruits, vegetables, fish, and whole grains, and detrimental effects for diets high in saturated fats, refined sugars, and processed foods.^10^,^11^ More recently, specific attention has turned towards dietary macronutrients, particularly protein, as a potential modulator of immune-inflammatory pathways relevant to asthma. Protein intake influences essential processes such as immune cell function, gut microbiota composition, and the production of inflammatory mediators and growth factors (e.g., IGF-1).^12^ Prior experimental work has revealed an association between high-protein diets and elevated type I hypersensitivity risk in OVA-sensitized mice. This heightened susceptibility is characterized by increased B-cell counts, elevated total serum protein and antigen-specific IgE levels, and a distinct Th1/Th2 polarization toward Th2 dominance.^13^ To date, there remains a paucity of research examining the relationship between dietary protein intake and asthma in children and adolescents. Mechanistically, high protein intake, especially from animal sources, may activate the mTOR pathway, promote Th2-mediated inflammation, and alter gut microbial ecology, potentially contributing to airway hyperreactivity.^12^

Despite growing evidence, critical knowledge gaps persist regarding the specific relationship between dietary protein intake and asthma in US children and adolescents. Existing studies are often limited by small sample sizes, cross-sectional designs, reliance on parental recall for both diet and asthma outcomes, lack of comprehensive assessment of protein sources (e.g., differentiating red meat, poultry, fish, dairy, legumes, nuts), and insufficient consideration of potential effect modifiers like obesity, race/ethnicity, and socioeconomic status.^11^ Furthermore, few large-scale, retrospective studies have specifically examined the association of diverse protein types and intake patterns with incident asthma diagnosis and longitudinal asthma control within the diverse US pediatric population. The heterogeneity in previous findings underscores the need for rigorous investigation.

Therefore, leveraging data from the National Health and Nutrition Examination Survey (NHANES), a nationally representative US database with rigorous dietary and health assessments, this study aims to investigate the association between total dietary protein intake, as well as intake from major animal and plant sources, and asthma prevalence among US children and adolescents. The authors hypothesize that higher total protein intake is positively associated with increased asthma prevalence. By comprehensively adjusting for key covariates and utilizing robust dietary methodology, this research seeks to provide novel insights into the role of dietary protein in childhood asthma, informing potential preventive and therapeutic nutritional strategies.

## Method

### Data sources and study population

**Figure 1:**
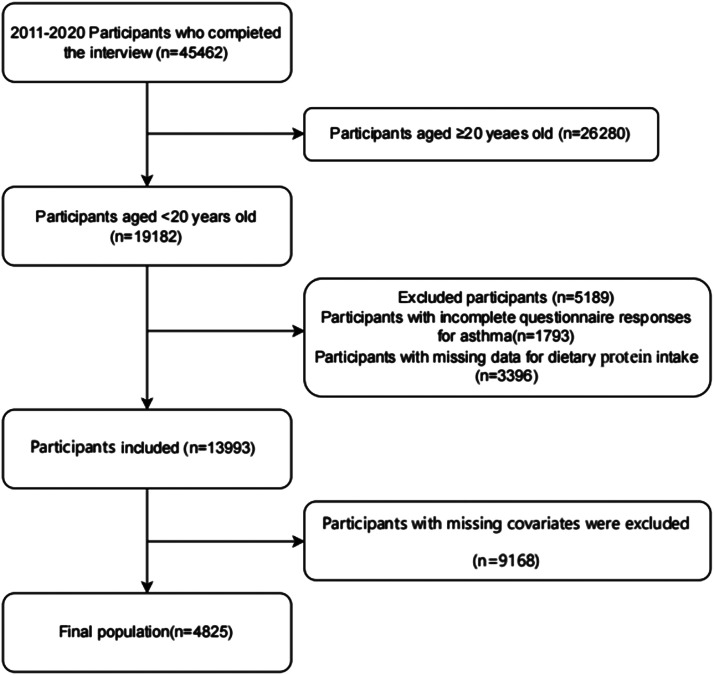
illustrates the thorough screening procedure.

This cross-sectional study utilized data from the 2011–2020 cycles of the National Health and Nutrition Examination Survey (NHANES). Administered biennially by the Centers for Disease Control and Prevention (CDC), NHANES provides comprehensive assessments of health and nutritional status within the United States civilian noninstitutionalized population.^14^ The survey employs a complex, multistage, stratified probability sampling design to ensure nationally representative estimates.^15^ Written informed consent was obtained from all participants, and the survey protocol received approval from the National Center for Health Statistics (NCHS) Research Ethics Review Board. Secondary analysis of de-identified data required no additional institutional review board approval.^2^ The authors excluded participants with incomplete dietary protein intake records or missing asthma diagnosis data. The final analytical cohort comprised individuals aged < 20 years who completed both demographic interviews and dietary assessments. Publicly accessible datasets are available through the NHANES online repository (http://www.cdc.gov/nchs/nhanes.htm) (Figure 1).

### Asthma assessment

To identify participants with asthma, the authors assessed their answers to the question, “Has a doctor or other health professional ever told you that you have asthma?” in the medical condition questionnaire. Those who replied “yes” were classified as asthmatic, whereas those who responded “no” were classified as non-asthmatic.

### Dietary protein intake

Dietary survey participants within NHANES provided detailed 24-hour dietary recalls documenting all consumed foods and beverages. Documentation followed standardized procedures outlined in the NHANES Dietary Interviewer's Manual. Nutrient quantification, including total protein intake (mg/day), The NHANES Nutritional Interview Manual is a comprehensive guide that meticulously explains the methodologies employed in nutritional surveys. Participants were then stratified into tertiles of protein intake (T1: lowest; T2: intermediate; T3: highest) based on mean daily consumption levels.

### Covariates

Several potential covariates were evaluated according to previous literature requirements,^16^, ^17^ including age, sex, race and ethnicity, family income, ratio of income to poverty (PIR), body mass index (BMI) category, mother smoking during pregnancy, mother’s age at birth, birth weight, white blood cell counts, red blood cell counts, eosinophil counts, serum folate, and daily intakes of vitamin C obtained through 24-h dietary recall interviews, as well as intakes of vitamin D, vitamin E, sugar, PIR was computed by dividing income(household or individual) by the poverty threshold for the survey year. Household income was divided into three classes based on PIR, according to a U.S. government report (20): low (PIR < 1.3), moderate (PIR: 1.3 − 3.5), and high (PIR > 3.5).^18^ Familial asthma predisposition was ascertained using the validated NHANES item: "Were blood relatives had asthma?" (Affirmative responses = positive status). Maternal smoking exposure, a recognized prenatal asthma risk factor was defined by binary response to the standardized query: "Did the biological mother smoke during pregnancy?" If the mother had answered “yes,” she was deemed to have smoked; if not, she was deemed to have not smoked throughout her pregnancy. Critical perinatal covariates included: Mother's age at childbirth (years), Birth weight (pounds; converted to grams for analysis), with both variables demonstrating established associations with pediatric asthma development. All operational definitions align with NHANES Demographic Variables Manual protocols.^2^

### Statistical analysis

The authors conducted a follow-up examination of the NHANES information. Categorical variables were expressed as percentages, and continuous variables were expressed as mean ± SD or median (IQR), depending on the data distribution. Firstly, the authors split the total amount of protein consumed into three equal halves. Subsequently, differences between groups were assessed using multivariable analysis. Three linear regression models were constructed:

Model 1:Adjusted for demographic characteristics (age, sex, race/ethnicity, poverty income ratio [PIR]).
Model 2: Incorporated additional adjustments for maternal smoking during pregnancy, family history of asthma, maternal age at delivery, birth weight, white blood cell count, red blood cell count, serum folate, and eosinophil count.
Model 3: Fully adjusted to ascertain the association between dietary protein intake and asthma, incorporating vitamin C, vitamin D, vitamin E, and total dietary sugar intake as covariates.

Furthermore, employing a three-knot restricted cubic spline (RCS), the authors examined the dose-response relationship between dietary protein intake and asthma, with covariate adjustment based on Model 3. To explore potential effect modification, subgroup analyses were performed, stratified by sex, age, PIR, race/ethnicity, maternal smoking during pregnancy, and family history of asthma. R statistical Software 4.2.2(http://www.R-project.org, The R Foundation) and Free Statistics software 2.1.1 were used for all studies. A two-sided P value of <0.05 was deemed statistically significant in the present investigation.

Continuous covariates with large numerical scales underwent magnitude scaling to enhance model stability and coefficient interpretability. Specifically, total dietary protein intake(continuous independent variable) was multiplied by 0.1, converting the unit to “per 0.1 mg/kg/day increment”. This linear transformation preserves the variable’s distributional properties while mitigating computational issues arising from extreme exponent values during logistic regression fitting.

## Results

### Baseline characteristics of participants

This analysis included 4825 US children and adolescents stratified by total protein intake tertiles (Q1 = lowest, Q3 = highest). Significant differences were observed across protein intake groups for several variables. Males comprised a progressively larger proportion across increasing tertiles (Q1:41.0 %, Q2:47.6 %, Q3:60.5 %, *p* < 0.001), while the inverse was true for females. Mean age was slightly higher in Q3 (10.9 ± 2.8 years) compared to Q1 (10.5 ± 2.9) and Q2 (10.3 ± 2.9) (*p* < 0.001). Racial/ethnic distribution did not differ significantly (*p* = 0.26). Nutritional biomarkers demonstrated strong positive associations with protein intake: vitamins C, D, E (all *p* ≤ 0.006), total sugar (*p* < 0.001), and serum folate (*p* < 0.001) levels increased across tertiles. White blood cell (WBC, *p* = 0.037) and red blood cell (RBC, *p* < 0.001) counts, eosinophil counts (*p* < 0.001), and BMI (21.1 ± 5.4 in Q3 vs 20.6 ± 5.6 in Q1, *p* = 0.023) also varied significantly. Socioeconomic status (PIR) showed a gradient, with lower PIR more prevalent in Q1 (48.3 %) and higher PIR in Q3 (21.3 %) (*p* < 0.001). Familial asthma prevalence (*p* = 0.072) and maternal smoking during pregnancy (*p* = 0.056) did not reach statistical significance across groups. Birth weight and maternal age at delivery were comparable (Table 1).

**Table 1:** Baseline characteristics of the study participants.

Variables Total (*n* = 4825)		Total protein intake (gm/day)			
		Q1 (*n* = 1608)	Q2 (*n* = 1608)	Q3 (*n* = 1609)	p
Sex, n ( %)					< 0.001
Male	2400 (49.7)	660 (41)	766 (47.6)	974 (60.5)	
Female	2425 (50.3)	948 (59)	842 (52.4)	635 (39.5)	
Age, Mean ± SD	10.6 ± 2.9	10.5 ± 2.9	10.3 ± 2.9	10.9 ± 2.8	< 0.001
Race and ethnicity, n ( %)					0.26
Mexican American	1026 (21.3)	329 (20.5)	336 (20.9)	361 (22.4)	
Other Hispanic	534 (11.1)	170 (10.6)	179 (11.1)	185 (11.5)	
Non-Hispanic White	1358 (28.1)	458 (28.5)	444 (27.6)	456 (28.3)	
Non-Hispanic Black	1209 (25.1)	431 (26.8)	415 (25.8)	363 (22.6)	
Other/Multiracial	698 (14.5)	220 (13.7)	234 (14.6)	244 (15.2)	
Weight at birth (pounds)	7.0 (6.0, 8.0)	7.0 (6.0, 8.0)	7.0 (6.0, 8.0)	7.0 (6.0, 8.0)	0.65
Mother’s age when born (years)	26.0 (22.0, 31.0)	26.0 (22.0, 31.0)	27.0 (22.0, 32.0)	26.0 (22.0, 31.0)	0.329
WBC (1000 cells/μL)	7.0 ± 2.1	6.9 ± 2.1	7.1 ± 2.1	7.0 ± 2.0	0.037
RBC (1000 cells/μL)	4.7 ± 0.4	4.7 ± 0.4	4.7 ± 0.4	4.7 ± 0.4	< 0.001
Serum folate (nmol/L)	53.9 ± 21.1	56.1 ± 22.8	54.3 ± 20.8	51.2 ± 19.2	< 0.001
Vitamin C (mg/day)	50.8 (20.1, 103.7)	36.2 (12.5, 86.0)	51.7 (21.6, 103.7)	67.3 (27.8, 123.1)	< 0.001
Vitamin D (mg/day)	4.2 (1.9, 7.2)	2.6 (0.7, 4.6)	4.5 (2.4, 7.2)	6.3 (3.5, 9.7)	< 0.001
Vitamin E (mg/day)	0.0 (0.0, 0.0)	0.0 (0.0, 0.0)	0.0 (0.0, 0.0)	0.0 (0.0, 0.0)	0.006
Sugar (mg/day)	116.2 ± 63.1	91.0 ± 49.5	115.0 ± 56.3	142.6 ± 70.7	< 0.001
Eosinophil counts (cells/μL)	0.2 (0.1, 0.3)	0.2 (0.1, 0.3)	0.2 (0.1, 0.3)	0.2 (0.1, 0.3)	< 0.001
Familial asthma, n ( %)					0.072
No	3174 (65.8)	1045 (65)	1093 (68)	1036 (64.4)	
Yes	1651 (34.2)	563 (35)	515 (32)	573 (35.6)	
PIR, n ( %)					< 0.001
Low	2143 (44.4)	776 (48.3)	706 (43.9)	661 (41.1)	
Medium	1726 (35.8)	555 (34.5)	566 (35.2)	605 (37.6)	
High	956 (19.8)	277 (17.2)	336 (20.9)	343 (21.3)	
Mother smoked when pregnant, n ( %)					0.056
No	4273 (88.6)	1401 (87.1)	1444 (89.8)	1428 (88.8)	
Yes	552 (11.4)	207 (12.9)	164 (10.2)	181 (11.2)	
BMI, Mean ± SD	20.8 ± 5.6	20.6 ± 5.6	20.7 ± 5.6	21.1 ± 5.4	0.023

BMI, body mass index; PIR, ratio of income to poverty; WBC, white blood cell count; RBC, red blood cell count; T, tertiles.

### Relationship between protein intake and asthma

Using multiple linear regression models, trend tests were run to investigate the relationship between total protein intake and asthma (Table 2). Total protein intake and asthma were shown to be positively associated; after controlling for the three models, the difference was statistically significant (*p* = 0.037). Once potential confounders were ruled out, participants’ overall protein intake — which was divided into three groups — continued to have a favorable correlation with asthma levels. Model 1: (minimally adjusted): OR = 1.30 (95 % CI: 1.08–1.55; *p* = 0.005). Model 2: (further adjusted): OR = 1.29 (95 % CI: 1.07–1.56; *p* = 0.01). Model 3(fully adjusted for covariates): OR = 1.26 (95 % CI: 1.01–1.55; *p* = 0.036). Total protein intake was higher in the second and third groups than in the people in the T1 (≤ 3.966). A significant dose-response relationship was confirmed by trend tests across all models (*p* < 0.05), with Model 3 yielding *p* = 0.037 (Table 2).

**Table 2:** Linear regression model analysis elucidates the correlation between total protein intake and asthma.

Quartile	OR(95 % CI)									
	No.	n( %)	crude	*P*-value	Model 1	*P*-value	Model 2	*P*-value	Model 3	*P*-value
Protein intake (gm/day)										
Q1 (≤ 3.966)	1608	276 (17.2)	1(Ref)		1(Ref)	0.152	1(Ref)		1(Ref)	
Q2 (3.967∼6.364)	1608	309 (19.2)	1.15 (0.96∼1.37)	0.132	1.14 (0.95∼1.37)	0.005	1.21 (1∼1.47)	0.063	1.18(0.97∼1.44)	0.097
Q3 (≥ 6.365)	1609	353 (21.9)	1.36 (1.14∼1.62)	0.001	1.3 (1.08∼1.55)	0.005	1.29 (1.07∼1.56)	0.01	1.26 (1.01∼1.55)	0.036
Trend. test	4825	938 (19.4)		0.001		0.005		0.01		0.037

Note:.

Model 1: adjusted for age, sex, race/ethnicity, BMI, PIR;.

Model2: Model1+ Mother smoked when pregnant, Mother’s age when born, Weight at birth, Familial asthma, WBC, RBC, Serum folate, eosinophil counts.

Model3: Model2+ Vitamin C, Vitamin D, Vitamin E, Suga.

### Dose-response relationship between total protein intake and asthma

**Figure 2:**
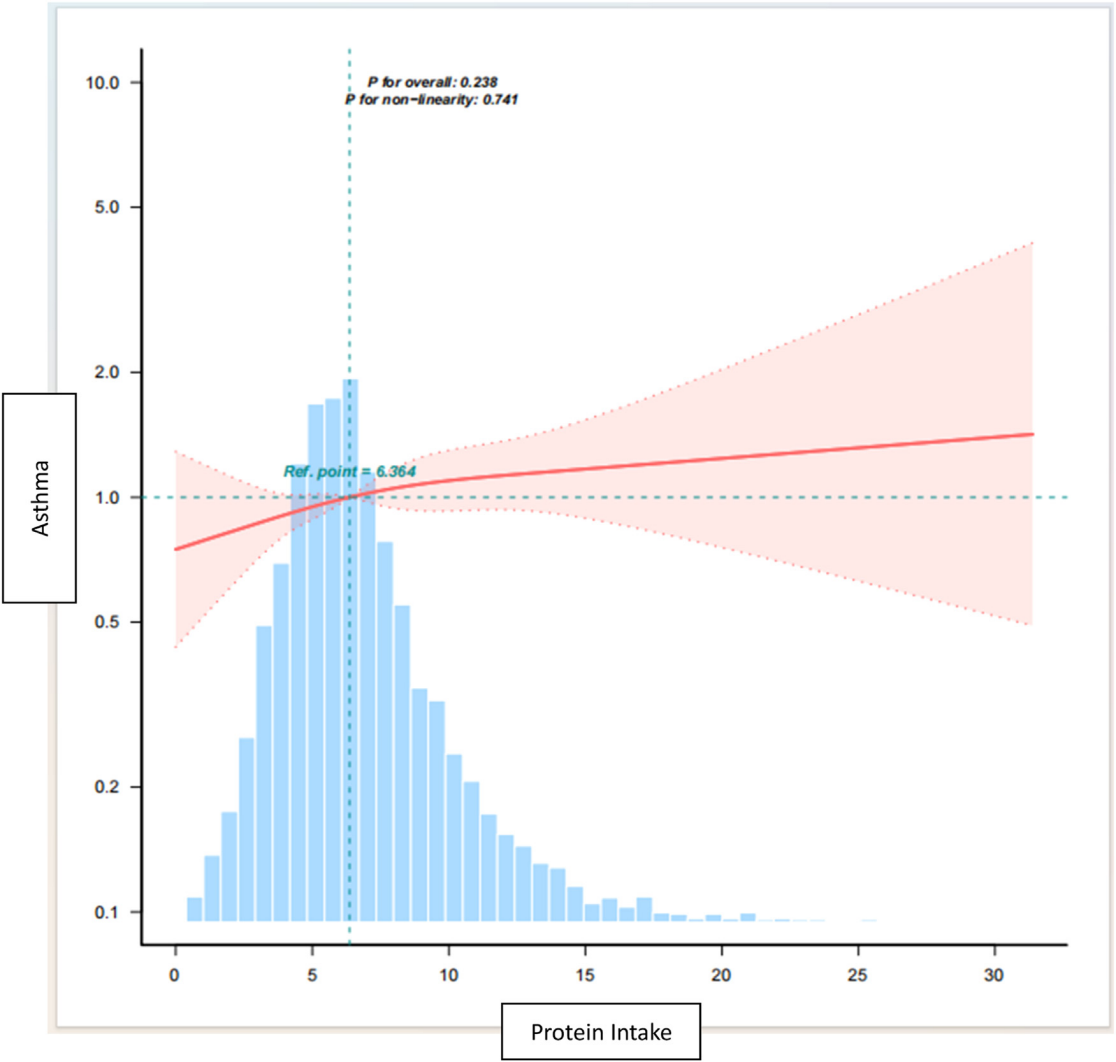
Dose -response relationship between total protein intake and asthma. Note: Lines represent adjusted slopes (solid lines) and 95 % confidence intervals (long dashed lines). Histograms represent the distribution of total protein intake in children and adolescents with asthma. Adjusted for age, sex, race/ethnicity, BMI Category, PIR, Mother smoked when pregnant, Mother’s age when born, Weight at birth, Familial asthma, WBC, RBC, Serum folate, Vitamin C, Vitamin D, Vitamin E, sugar, eosinophil counts. Abbreviations: PIR, ratio of income to poverty; BMI, body mass index; WBC, white blood cell count; RBC, red blood cell count.

The restricted triple spline analyses, adjusted for variables consistent with model 3, revealed a significant linear relationship between total protein intake and asthma (Figure 2, *p* = 0.741). The estimated dose-response curves also supported this finding. Increased intake of dietary protein is associated with an upward trend in the risk of asthma.

### Stratified analysis based on covariates

**Figure 3:**
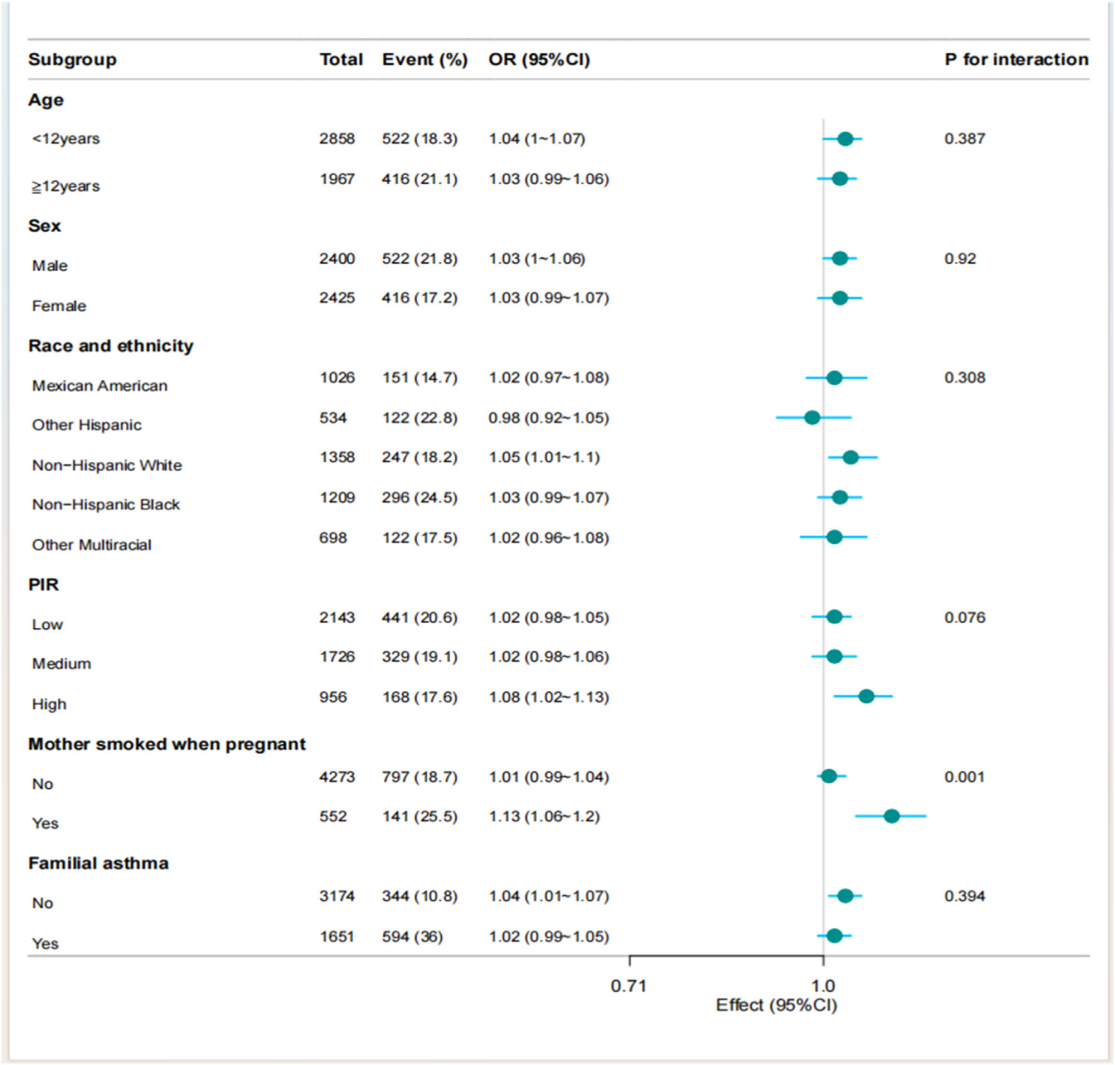
Forest plot of multivariate logistic analysis between total protein intake and asthma. PIR, ratio of income to poverty.

To assess the robustness of multivariate regression findings, the authors evaluated the association between total protein intake and asthma across key demographic and exposure-defined subgroups. Stratified analyses by ratio of income to poverty (PIR), sex, age group, and family asthma history revealed no significant interaction effects. However, significant effect modification was identified for maternal smoking during pregnancy (*p* = 0.001), indicating differential associations according to prenatal smoke exposure status. These findings necessitate further validation in independent cohorts to establish causal inference pathways (Figure 3).

## Discussion

Through an analysis of NHANES data from 2011 to 2020, this study looked at the link between total dietary protein intake and asthma in children and adolescents in the US. According to the findings, there was a substantial and positive correlation between total dietary protein intake and asthma. Furthermore, a linear correlation was observed between the overall amount of protein consumed in the diet and the number of asthma cases, as verified by the results of the restricted triple spline test. The present study demonstrates a significant positive association between higher dietary protein intake and asthma prevalence among US children and adolescents, persisting after comprehensive adjustment for demographic, socioeconomic, and lifestyle confounders. The present findings align with emerging evidence suggesting a complex role of dietary protein in pediatric respiratory health. While traditional nutritional guidelines emphasize adequate protein for growth, these observation of a dose-dependent relationship, particularly with higher intake tertiles (≥ 6.365 mg/day), warrants careful consideration of potential adverse effects at supra-physiological levels.

Approximately 50 % of mild-to-moderate asthma cases and a considerable subset of severe asthma are primarily mediated by Th2-dependent inflammation. Emerging evidence indicates contributory roles for non-inflammatory processes in asthma pathogenesis.^19^ Asthmatic patients demonstrate hypersensitivity to triggers (e.g., allergens); subsequent exposures induce inflammatory cascades and reactive oxygen species (ROS) release, consequently elevating oxidative stress susceptibility.^20^ The association between asthma symptomatology and dietary protein intake remains inadequately investigated. Impairment of lung function.^21^ Current evidence suggests that higher protein consumption may exacerbate asthma.^22^,^23^

Protein-rich foods provide indispensable amino acids alongside saturated and unsaturated lipids, vitamins, minerals, and bioactive phytochemicals. Chronic excessive intake, however, disrupts metabolic homeostasis by promoting positive energy balance. Meta-analyses of prospective cohorts demonstrate that sustained high-protein consumption correlates with increased incidence of obesity, type 2 diabetes, cardiovascular disease, and all-cause mortality. This association is mechanistically explained through sequential metabolic conversion: surplus amino acids undergo hepatic gluconeogenesis to glucose, with subsequent de novo lipogenesis contributing to BMI elevation. The "early protein hypothesis" ^24^ further elucidates this process: protein intake exceeding physiological requirements augments insulin and insulin-like growth factor-1 (IGF-1) secretion, thereby inducing adipocyte differentiation and lipogenesis while suppressing lipolysis — ultimately driving adipose tissue expansion,^25^ potentially via obesity-related pathways given its established role in adversely impacting respiratory health through multiple mechanisms: diminished lung volumes,^26^ impaired pulmonary function,^27^ and aggravated symptom burden including wheezing.^28^

This work deliberately focuses on the previously unexamined association between dietary protein intake and pediatric asthma. Analysis incorporated a nationally representative cohort of asthmatic children/adolescents, with adjustment for multiple confounders. The persistent dose-dependent gradient in odds ratios across sequentially adjusted models further corroborates the robustness of the present findings.

### Limitations and implications

This study has several notable limitations: a) Although asthma diagnosis was established, disease severity, atopic status, and lung function parameters were not evaluated; b) Other macronutrients with potential confounding effects — notably dietary fiber, total caloric intake, and total fat consumption—were not assessed. Nevertheless, this nationally representative study highlights a potential modifiable dietary risk factor for pediatric asthma. Future longitudinal and mechanistic studies should delineate whether specific protein subtypes (dairy, red meat, plant proteins) or amino acid profiles drive this association and explore interventions optimizing protein intake thresholds during critical developmental windows.

## Conclusion

Based on a robust correlation demonstrated between total dietary protein intake and asthma in US children and adolescents, this study contributes a novel perspective to the holistic management of asthma. Restricting overall protein consumption may represent a critical component of nutritional therapy for pediatric asthma patients, potentially enhancing quality of life and improving disease control. Further investigations are warranted to validate these findings and investigate their clinical translation.

### Data availability statement

The datasets supporting this study are available from the corresponding author upon reasonable request.

## Funding

This research did not receive any specific grants from funding agencies in the public, commercial, or not-for-profit sectors.

## Authors’ contributions

(1) The conception and design of the study. (Lingyu Li, Peipei Tian). (2) Acquisition of data.(Lingyu Li, Yanmei Teng). (3) Analysis and interpretation of data. (Lingyu Li, Rui Wang). (4) Drafting the article or revising it critically for important intellectual content. (Lingyu Li, Qiuyu Ma). (5) Final approval of the version to be submitted. (Lingyu Li, Mingxiao Zhang). All authors read and approved the final manuscript.

## Ethics approval

The NHANES protocol obtained approval from the National Center for Health Statistics (NCHS) Ethics Review Committee, with informed consent secured from all participants prior to enrolment. No further institutional review board approval was necessary for this secondary analysis. The procedures used in this study adhere to the tenets of the Declaration of Helsinki.

## Conflicts of interest

The authors declare no conflicts of interest.
